# Risk of developing second malignant neoplasms in patients with neuroblastoma: a population study of the US SEER database

**DOI:** 10.1186/s13014-021-01943-x

**Published:** 2021-11-27

**Authors:** Hongnan Zhen, Hui Guan, Jiabin Ma, Wenhui Wang, Shen Jing, Zheng Miao, Fuquan Zhang, Zhikai Liu

**Affiliations:** Department of Radiation Oncology, Peking Union Medical College Hospital, Chinese Academy of Medical Sciences & Peking Union Medical College, No. 1 of Shuaifuyuan Road, Dongcheng District, Beijing, 100730 China

**Keywords:** Neuroblastoma, Second malignant neoplasms, Survival rate, Radiotherapy, Chemotherapy

## Abstract

**Background:**

Neuroblastoma is a common extracranial malignant tumor in children. Its main treatment modality is a combination of chemotherapy, radiotherapy, and surgery. Given the advances in chemotherapy regimens and the widespread use of bone marrow transplantation over the decades, there has been improvement in treatment efficacy, which has led to prolonged patient survival. Accordingly, long-term complications have become a growing concern among physicians and patients. This study aimed to analyze the survival rate of patients with neuroblastoma and the risk factors for developing second malignant neoplasms (SMNs).

**Methods:**

The SEER 18 Regs (1973–2015) and SEER 9 Regs (1973–2015) data of the surveillance, epidemiology, and end results (SEER) database of the US National Cancer Institute were adopted for survival and SMN analysis.

**Results:**

The 5-, 10-, and 20-year overall survival rates of patients with neuroblastoma were 67%, 65%, and 62%, respectively. Among 38 patients with neuroblastoma who presented with SMNs, those with abdomen as the primary site accounted for the majority (63.2%), followed by those with thorax (26.3%) and other sites (10.5%). SMNs occurred more commonly in non-specific neuroblastoma (incidence: 0.87%) than ganglioneuroblastoma (incidence: 0.3%). Compared with the general population, the risk of SMN is significantly higher (SIR = 4.36). The risk of developing SMNs was significantly higher in the digestive system (SIR = 7.29), bones and joints (SIR = 12.91), urinary system (SIR = 23.48), brain and other nervous systems (SIR = 5.70), and endocrine system (SIR = 5.84). Multivariate analysis revealed that the year of diagnosis (OR = 2.138, 95% CI = 1.634–2.797, *p* < 0.001) was the only independent risk factor for developing SMNs.

**Conclusion:**

This study identifies the risk factor for developing SMNs in patients with neuroblastoma, which could facilitate individualized screening for high-risk patients, to allow early diagnosis and treatment of SMNs.

## Background

Neuroblastoma originates from the adrenal medulla or the paravertebral sympathetic nervous system. It is the most common extracranial solid tumor in children. According to the National Cancer Institute surveys in the United States, the incidence of neuroblastoma was 10.54/1000,000 (between 1975 and 2009). Neuroblastoma is a strongly heterogeneous tumor in children aged < 15 years. Some tumors can spontaneously disappear without treatment; however, most are insidious and have metastasized to the whole body at the time of diagnosis, with rapid progression ultimately leading to death. International multi-center collaboration of more than 30 years has facilitated an increase in the 5-year survival rate from 46% in 1974–1989 to 71% in 1999–2004. This is mainly attributed to the widespread adoption of risk-based stratification of therapy, including decreasing and increasing the treatment intensity for low-to intermediate-risk and high-risk patients [1], respectively, as well as performing autologous stem cell transplantation and metaiodobenzylguanidine (MIBG) treatment. Given the improvement in diagnosis and treatment and the concomitant prolongation of patient survival, long-term complications, represented by second malignant neoplasms (SMNs), have been increasingly attracting attention from physicians and patients. Most patients require postoperative chemotherapy with high-risk patients even needing radiotherapy; contrastingly, few low-risk patients only require postoperative follow-up observation. Radiotherapy was previously considered the main causative factor of SMNs; however, an increasing number of patients who have only undergone surgical treatment without radiotherapy are developing SMNs [2, 3]. This suggests that endogenous factors (mainly genetic and environmental factors) may be involved in the SMN development. In terms of incidence, neuroblastoma is a rare disease and mostly occurs in children and adolescents; it is difficult to study the incidence of SMN through single-center data. Therefore, this study aimed to use data from the Surveillance, Epidemiology, and End Results (SEER) database to investigate the risk factors for developing SMNs after neuroblastoma treatment and explore their impact on patient prognosis.

## Methods

Information of patients with neuroblastoma (including other treatment information) from 1973 to 2015 were retrieved from the SEER database using the software SEER*Stat 8.3.6.1 The inclusion criteria were as follows: (1) patients with ICD-O-3 Hist/behave = neuroblastoma (ICD-O-3 = 9500) or ganglioneuroblastoma (ICD-O-3 = 9490); (2) patients with clear pathological classification and staging; (3) age of diagnosis less than 20 years, and (4) patients with complete follow-up information. The exclusion criteria were as follows: (1) patients diagnosed by autopsy; (2) primary site of the tumor is the central nervous system (CNS), and (3) patients with incomplete follow-up information. The survival time investigated in this study was the overall survival (OS) time. SMNs are defined as other malignant tumors appearing at least 2 months after neuroblastoma diagnosis. The standardized incidence ratios (SIRs) of SMNs were computed and analyzed using SEER*Stat 8.3.6.1; further, the corresponding 95% confidence level (CI) was reported. We collected information regarding age at diagnosis, gender, race, pathological type, degree of pathological differentiation, year of diagnosis, primary lesion site, whether radiotherapy was received, whether chemotherapy was received, survival time, and survival status. Authorization was obtained through the SEER website and data were retrieved from the SEER database, with no additional ethical approval being required.

### Statistical analyses

Statistical analyses were performed using SPSS 22.0 (SPSS Inc, Chicago, IL). Statistical significance was set as *p* ≤ 0.05. Specifically, the Kaplan–Meier method was used to estimate patient survival time. Univariate and multivariate regression analyses were performed using the Log-Rank test and Cox proportional hazards model, respectively. Independent risk factors for developing SMNs were analyzed using logistic regression. Independent risk factors for death were initially identified using univariate analysis; among them, those with *p* ≤ 0.1 were included in the multivariate analysis. The calculation of SIRs are calculated by SEER-stat software (compared with age-matched general population).

## Results

### Clinical characteristics of patients

This study enrolled 4338 patients, with 3667 patients pathologically-diagnosed with neuroblastoma and 671 with ganglioneuroblastoma. The abdomen (n = 3019) was the most common primary site, followed by the chest (n = 773). Males (52.9%) and females (47.1%) had roughly the same incidence rate. The incidence rates of patients aged < 1 year, 1–5 years, and 6–19 years were 34.4%, 54.1%, and 11.5%, respectively. Table 1 shows the clinical characteristics of all patients with neuroblastoma, including those with SMNs. Among 38 patients with neuroblastoma who presented with SMNs, those with abdomen as the primary site accounted for majority (63.2%), followed by those with thorax (26.3%), and other site (10.5%). SMNs occurred more commonly in non-specific neuroblastoma (incidence: 0.87%) than ganglioneuroblastoma (incidence: 0.3%). Compared with the general population, the risk of SMN is significantly higher (SIR = 4.36). The risk of developing SMNs was significantly higher in the digestive system (SIR = 7.29), bones and joints (SIR = 12.91), urinary system (SIR = 23.48), brain and other nervous systems (SIR = 5.70), and endocrine system (SIR = 5.84).

**Table 1:** Demographic and clinical characteristics of patients with neuroblastoma, SEER 18

Variables	Categories	Cohort (%)	
		All NB patients (n = 4338)	SMN (n = 38)
Sex	Male	2294 (52.9)	19 (50.0)
	Female	2044 (47.1)	19 (50.0)
Age at diagnosis (first primary) (years)	< 1	1493 (34.4)	14 (36.8)
	1–5	2347 (54.1)	18 (47.4)
	6–19	498 (11.5)	6 (15.8)
Year of diagnosis	1973–1979	303 (7.0)	5 (13.2)
	1980–1989	506 (11.7)	18 (47.4)
	1990–1999	750 (17.3)	5 (13.2)
	2000–2009	1739 (40.1)	9 (23.7)
	2010–2015	1040 (24.0)	1 (2.6)
Primary Site	Abdomen	3019 (70.7)	24 (63.2)
	Thorax	773 (18.1)	10 (26.3)
	Others	478 (11.2)	4 (10.5)
Histologic type	Neuroblastoma, NOS	3667 (84.5)	36 (94.7)
	Ganglioneuroblastoma	671 (15.5)	2 (5.3)
Race	White	3410 (79.6)	30 (78.9)
	Black	528 (12.3)	5 (13.2)
	Other	348 (8.1)	3 (7.9)
Radiotherapy	Yes	1085 (25.0)	19 (50.0)
	No/unknown	3253 (75.0)	19 (50.0)
Chemotherapy	Yes	2829 (65.2)	28 (73.7)
	No/unknown	1509 (34.8)	10 (26.3)

SEER 18, surveillance, epidemiology, and end results program 18; NB, Neuroblastoma; SMN, second malignant neoplasm; NOS, not otherwise specified

### Survival of patients with neuroblastoma

The 5-, 10-, and 20-year OS rates of the entire neuroblastoma cohort were 67%, 65%, and 62%, respectively. Independent risk factors for death were initially identified using univariate analysis; among them, those with *p* ≤ 0.1 were included in the multivariate analysis. Univariate analysis identified the following significant factors: age (*p* < 0.001), year of diagnosis (*p* < 0.001), histologic type (*p* < 0.001), primary site (*p* < 0.001), chemotherapy (*p* < 0.001), radiotherapy (*p* < 0.001), degree of pathological differentiation (*p* < 0.001), gender (*p* = 0.004), and race (*p* = 0.007). SMNs (*p* = 0.175) were not a risk factor for mortality. Factors with *p* ≤ 0.1 were included in a Cox proportional hazards model, which identified the following independent risk factors for patient OS: age (OR = 2.099,95% CI = 1.807–2.439, *p* < 0.001), year of diagnosis (OR = 5.710,95% CI = 3.225–10.110, *p* < 0.001), degree of pathological differentiation (OR = 1.561,95% CI = 1.352–1.801, *p* < 0.001), primary site (OR = 1.829,95% CI = 1.285–2.602, *p* < 0.001), chemotherapy (OR = 2.864,95% CI = 2.048–4.005, *p* < 0.001).Radiotherapy (*p* = 0.08) is not a risk factor for mortality.

### Risk factors for developing SMNs

**Fig. 1:**
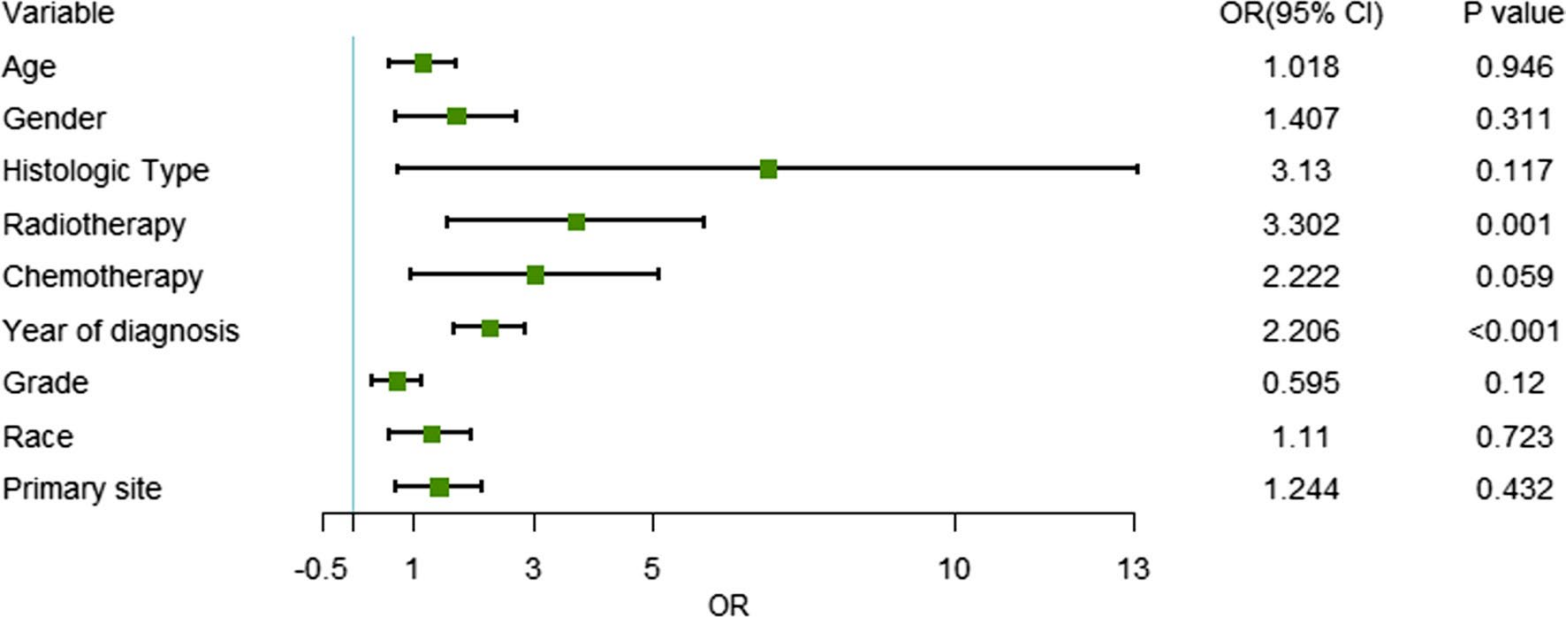
The correlationship between risk factors for SMN in neuroblastoma

A total of 38 patients were diagnosed with SMNs. The SIR of all the patients was 4.36 (95% CI = 3.08–5.97, *p* < 0.05). Univariate analysis showed that the year of diagnosis (OR = 2.206, 95% CI = 1.695–2.874, *p* < 0.001), radiotherapy (*p* = 0.001), and chemotherapy (*p* = 0.059) were significantly correlated with the incidence of SMNs (Fig. 1). Multivariate analysis showed that the year of diagnosis (OR = 2.138, 95% CI = 1.634–2.797, *p* < 0.001) was the only independent risk factors for developing SMNs. The result showed that patients with earlier year of diagnosis were more likely to develop SMN. The SIR of SMN gradually increased from 3.44 in the 1970s to 8.14 in the 2000s (*p* < 0.05). Radiotherapy (*p* = 0.109) and chemotherapy (*p* = 0.082) were not independent risk factors for developing SMNs.

## Discussion

This population-based cohort study suggests that the risk of SMNs is higher in patients with neuroblastoma than in the normal population, with the year of diagnosis affecting the incidence of SMNs. There have been several changes in neuroblastoma treatment since the 1970s, including risk-based stratification of therapy (where the treatment intensity for low- to intermediate-risk patients and high-risk patients is decreased and increased, respectively), autologous transplantation, and MIBG treatment. Update of treatment concept and advancement of treatment technology have led to a significant increase in survival rates, which has allowed observation of long-term side effects represented by SMNs in long-term survivors.

Our findings indicate that the year of diagnosis is an independent risk factor for the incidence of SMNs in patients with neuroblastoma. The result showed that patients with an earlier year of diagnosis are more likely to develop SMN, which may be associated with the extension follow-up time and evolution of treatment during the period. High-risk patients are recommended to receive higher-intensity radiotherapy and chemotherapy. Chemotherapy-related hematological SMN usually appeared within a few years after treatment. This has led to a significant increase in the risk of total SMN. However, due to the limitation in the number of cases in the SMN cohort, this view still needs more long-term follow-up and a larger sample size for verification.

Our study shows that the SMN incidence rate of the entire cohort is 0.88%, which is similar to the incidence of several other neuroblastoma SMN cohorts [2, 4], which is consistent with the results of the Childhood Cancer Survivor Study (CCSS). Studies have shown that the probability of neuroblastoma survivors developing SMNs greatly increases with an increase in survival time. However, the incidence of SMNs does not tend to plateau over time [1, 5–7]. The results of the above studies indicated that the time to SMN occurrence varied according to the pathological type of SMN. The majority of lymphatic and hematopoietic SMNs occur about 5 years after treatment; sarcoma occurs between 5 and 15 years after treatment; and carcinoma usually occurs after 15 years of treatment. With the follow-up time increasing, SMNs are expected to have higher incidence rates.

Our study reveals that radiotherapy is not the independent risk factor for developing SMNs, although most studies have indicated the carcinogenic role of radiotherapy in neuroblastoma treatment [4, 7–12]. Compared to other childhood tumors, radiotherapy doses to treat neuroblastoma are low, and low-dose radiation is more likely to cause radiation-related carcinogenesis. However, the aforementioned conclusions may only apply to traditional radiotherapy dominated by external irradiation. We found in univariate analysis that radiotherapy was an independent risk factor for SMNs (*p* = 0.001), but multivariate analysis showed that radiotherapy was not a risk factor for SMNs (*p* = 0.109). 18 of the 38 patients in our SMN cohort had received radiotherapy (47.3%), but we were unable to obtain detailed information such as the field of radiotherapy and the site of the SMNs to assess whether SMN occurred in the radiation field. Another reason that may affect the statistical results is that the SMN cohort has a small sample size, which makes it impossible to calculate statistical differences. Some study showed radiotherapy may not play a role in the occurrence of SMN [3, 13] mainly because they found that a considerable amount of SMN did not occur in the radiation field, and a small number of SMN patients did not receive radiotherapy; hence, there is insufficient evidence to prove that radiotherapy is implicated in the occurrence of SMN. Another point worth noting is that the wide application of three-dimensional conformal radiotherapy technology (3D-CRT) and intensity-modulated radiotherapy (IMRT) over the past decades has improved the accuracy of treatment and reduced the volume and dose to normal tissues surrounding the radiation field. This may reduce the possibility of SMN occurring around the radiation field. MIBG, which can be broadly considered as a type of radiotherapy, has also been widely applied in neuroblastoma treatment. However, many studies have shown that MIBG is not a risk factor for SMN. Moreover, the post-treatment incidence of SMNs does not vary with the increasing treatment times and radiation dose [3, 13].

Our study indicate that chemotherapy is not an influencing factor of SMNs, which is inconsistent with other reports [4, 8], and contrary to the current mainstream view. The current mainstream view is that chemotherapy is a risk factor for SMNs, especially cytotoxic drugs represented by alkylating agents and topoisomerase inhibitors, which can cause hematological SMNs such as acute myeloid leukemia (AML). Previously, intermediate-risk patients have a significantly higher risk of developing acute myelogenous leukemia even after receiving chemotherapy with low-dose alkylating agents and topoisomerase inhibitors [1–3, 7]. However, there is no clear evidence on whether these drugs can cause SMN for sarcoma and cancer. For example some SMN also appeared in patients receiving low-dose chemotherapy [2], and SMN caused by chemotherapy is not dose-dependent, which deserves attention [3]. Since the late 1990s, autologous stem cell transplantation has gradually become the standard of care for high-risk neuroblastoma. Studies have shown that the incidence of SMNs in patients with neuroblastoma receiving autologous stem cell transplantation therapy increased from 1.04% at 5 post-transplantation years to 2.6% at 10 post-transplantation years. Moreover, the incidence rate tends to continue to increase with an increasing follow-up duration [7]. Notably, low-risk patients usually only receive surgical therapy; however, they still have a significantly higher probability of developing SMNs than expected, which suggests that genetic factors may be crucially involved in the incidence of SMNs [14].

Additionally, patients with relapsed and refractory neuroblastoma are more likely to develop SMNs, which could be associated with genetic factors and higher-intensity chemoradiotherapy [13]. Another example that suggests genes likely playing a role in the development of SMN in patients with neuroblastoma is a SMN cohort had a family history of cancer, but these families did not meet the diagnostic criteria for Li-Fraumeni syndrome (LFS) [1]. LFS is a chromosomal dominant genetic disease, which is related to the mutation of the tumor suppressor gene *TP53*. LFS can cause various cancers, including breast cancer, brain tumors, sarcomas and other cancers. It usually has a clear family history, and most of it occurs at a young age. The probability of aggressive malignant tumors in high-risk family members before the age of 30 is as high as 50%. Osteosarcoma is a common SMN type after childhood tumor treatment, and is related to radiotherapy and chemotherapy but Ewing's sarcoma is not. A patient with Ewing's sarcoma was found in his SMN cohort, which is a rarer type of SMN pathology, meaning that genetic factors, rather than therapeutic factors such as radiotherapy and chemotherapy, may have caused the occurrence of this SMN. Another study found that multiple gene mutations exist in SMN patients with neuroblastoma [14], but due to the limitation in the number of cases, no statistical significance was found. The above results all indicate that genetic factors cannot be ignored in the pathogenesis of SMN and need to be further studied.

This study has several limitations. First, the treatment information in the SEER database is incomplete; moreover, there is missing key information, including disease stage, radiotherapy dose and genetic information, which impedes deeper analysis, although our study showed that age, year of diagnosis, degree of pathological differentiation, primary site, chemotherapy, and radiotherapy are independent risk factors for the prognosis of patients with neuroblastoma. We assumed that radiotherapy was performed in patients with high-risk disease, which may be a bias in interpreting the results. Neuroblastoma COG (Children's Oncology Group) risk stratification is mainly based on MYCN gene amplification, age, INSS (International Neuroblastoma Staging System) staging and other factors. Because the SEER database lacks MYCN gene amplification, INSS staging information, the current data cannot be used for risk stratification. Second, the SEER database does not represent the entire US patient population, which may affect the assessment of the actual incidence of SMNs in patients with neuroblastoma. Furthermore, the patient data included in the database cover a large time span with the risk stratification and treatment strategies of neuroblastoma having significantly changed several times since the 1970s. Therefore, the current stratification information and treatment regimens may not match those of past decades, which could introduce bias into the data. Nevertheless, this study revealed some characteristics regarding the incidence of SMNs in patients with neuroblastoma, which provide support for screening of long-term survivors, as well as early detection and intervention of SMNs.

## Conclusion

This study offers data on the SMNs of patients with neuroblastoma, indicating the risk factors for SMNs, which provide insight for neuroblastoma survivors and their physicians during follow-ups.

## Data Availability

The raw data supporting the conclusions of this article will be made available by the authors, without undue reservation.

## Abbreviations

SMNs: Second malignant neoplasms
SEER: Surveillance, epidemiology, and end results
SIRs: Standardized incidence ratios
MIBG: Metaiodobenzylguanidine
CNS: Central nervous system
OS: Overall survival
CI: Confidence interval
CCSS: Childhood Cancer Survivor Study
